# Establishing adherence–concentration–efficacy thresholds of TDF–FTC pre-exposure prophylaxis for HIV prevention in African women: a protocol for the Women TDF–FTC Benchmark Study

**DOI:** 10.3389/frph.2024.1325257

**Published:** 2024-05-27

**Authors:** Linxuan Wu, Matilda Saina, Clare Brown, David Chege, Deborah Donnell, David V. Glidden, Kenneth Ngure, Nelly R. Mugo, Nina Akelo, Torin Schaafsma, Peter L. Anderson, Kenneth K. Mugwanya

**Affiliations:** ^1^ Department of Global Health, University of Washington, Seattle, WA, United States; ^2^ Department of Epidemiology, University of Washington, Seattle, WA, United States; ^3^ Center for Clinical Research, Kenya Medical Research Institute, Nairobi, Kenya; ^4^ Clinical Trials Research Laboratory, Kenyatta National Hospital, Nairobi, Kenya; ^5^ Vaccine and Infectious Disease Division, Fred Hutchinson Cancer Center, Seattle, WA, United States; ^6^ Department of Epidemiology and Biostatistics, University of California San Francisco, San Francisco, CA, United States; ^7^ School of Public Health, Jomo Kenyatta University of Agriculture and Technology, Nairobi, Kenya; ^8^ Department of Pharmaceutical Science, University of Colorado Denver, Aurora, CO, United States

**Keywords:** HIV pre-exposure prophylaxis, cisgender women, adherence, pharmacokinetics, efficacy

## Abstract

**Background:**

Oral pre-exposure prophylaxis (PrEP) using co-formulated emtricitabine (FTC) and tenofovir disoproxil fumarate (TDF) is a potent HIV prevention method for men and women, with its efficacy highly dependent on adherence. A pivotal HIV efficacy study combined with a directly observed pharmacological study defined the thresholds for HIV protection in men who have sex with men (MSM), which are the keys to PrEP promotion and development of new PrEP agents. For African women at risk for HIV and belonging to a priority group considered due to disproportionately high incident HIV infections, the variable adherence in PrEP clinical trials and the limited pharmacologic data have resulted in a lack of clarity about the PrEP adherence required for HIV protection. We propose a study to quantify the adherence–concentration–efficacy thresholds of TDF/FTC PrEP among African cisgender women to inform decisions about optimal PrEP dosing and adherence for HIV protection.

**Methods:**

We randomized 45 low-risk HIV-uninfected African women, aged 18–30 years old, to directly observe the TDF/FTC PrEP of two, four, or seven doses per week for 8 weeks. A complementary age-matched pregnant women cohort at high risk of HIV, who will receive seven doses per week, was recruited (*N* = 15) with the primary aim of establishing benchmark concentrations in dried blood spots and peripheral blood mononuclear cells. Plasma, whole blood (WB), urine, hair, vaginal fluid, and vaginal tissue (non-pregnant women only) were archived for future testing. Drug concentrations were measured using methods validated for each biological matrix. Pharmacokinetic models were fitted to drug concentrations to quantify concentration–adherence thresholds. To define the drug concentrations associated with HIV protection, we applied the newly defined thresholds from the primary pharmacologic trial to the subset of women randomized to TDF/FTC or TDF in the Partners PrEP Study with the drug concentration assessed in plasma and WB samples. Multiple imputation was used to construct a data set with drug concentrations at each visit when an HIV test was performed for the entire cohort, replicating the work for MSM.

**Discussion:**

The proposed study generated the first African women-specific TDF–PrEP adherence–concentration–efficacy thresholds essential for guiding the accurate interpretation of TDF/FTC PrEP programs and clinical trials of novel HIV prevention products using TDF/FTC as an active control.

**Clinical Trial Registration:**

ClinicalTrials.gov, identifier (NCT05057858).

## Background

Tenofovir disoproxil fumarate/emtricitabine (TDF/FTC) oral daily pre-exposure prophylaxis (PrEP) is highly effective in reducing the HIV acquisition risk when taken as prescribed (1, 2). In 2022, more than 77% of new infections in sub-Saharan Africa (SSA) occurred among young cisgender women, who are more than three times as likely to acquire HIV than their male peers (3). HIV incidence rates are persistently high among African cisgender women during pregnancy (4). Thus, African cisgender women are a priority group to receive HIV prevention packages, including PrEP.

PrEP effectiveness highly depends on adherence, and pharmacologic measures of drug exposure are critical to quantifying the adherence levels and interpreting the results obtained from PrEP studies and programs (5–7). For men who have sex with men (MSM), adherence benchmark concentrations for tenofovir–diphosphate (TFV–DP) in dried blood spots (DBSs) with directly observed therapy (DOT) dosing have been established from studies conducted in United States (US) populations (8). Applying these adherence benchmarks to the iPrEx trial HIV seroconversions helped define the adherence frequency associated with HIV protection for MSM (i.e., two, four, and seven doses per week for 84%, 99%, and 99% HIV risk reduction, respectively) (9–11). Currently, these US male-derived adherence–concentration–efficacy thresholds are being applied to studies of African women taking PrEP. However, the adherence–concentration–efficacy thresholds derived in US MSM may not directly extrapolate to African cisgender women, including during pregnancy because of the biological and pharmacological differences among these populations and the different modes of HIV transmission.

Data from observational and controlled studies indicated different adherence–concentration findings for women (12–16). Large PrEP studies among African cisgender women showed that only 62% and 44% of individuals taking four or more and six or more doses per week, respectively, were correctly identified based on daily medication electronic adherence monitoring (i.e., medication event monitoring systems, MEMS) using US-derived TFV–DP thresholds (12). Furthermore, pregnancy lowered the concentrations of TFV and TFV–DP by 58% and 27%, respectively, among HIV-uninfected African women continuing PrEP during pregnancy (13). Similarly, the IMPAACT 2009 study of DOT PrEP dosing among pregnant and postpartum African women showed DBS TFV–DP levels in pregnant women to be nearly 30%–40% lower than those in postpartum women (14). However, the clinical significance of these changes remains unknown because none of the studies measured the TFV–DP levels in peripheral blood mononuclear cells (PBMCs), which are targets of HIV infections.

There is still uncertainty about the adherence requirement for HIV protection for men and women (17–19). An analysis of HPTN 083 and HPTN 084 suggested that men reached 99% efficacy with two or more doses per week on average, whereas women reached 99% efficacy only at seven doses per week on average, but that analysis was limited by the small person-time in each adherence strata, and the 95% confidence intervals for each adherence level overlapped between men and women (17). Similarly, a large pooled analysis of 11 real-world oral PrEP studies suggested that cisgender women who maintained adherence levels of at least four doses per week of oral TDF/FTC PrEP achieved 90% protective effectiveness against HIV, but only 40% of women in the 11 studies achieved this adherence level (18). Moreover, a recent modeling study indicated that systemic, but not local tissue (vaginal, colorectal), drug concentrations highly predicted the TDF/FTC PrEP efficacy against HIV infection (19). Taken together, data on the true and achievable effectiveness of oral TDF/FTC PrEP in women are still needed. To address these gaps, we propose determining the adherence–concentration–efficacy thresholds of TDF/FTC PrEP among African cisgender women.

## Methods

### Study design

**Figure 1:**
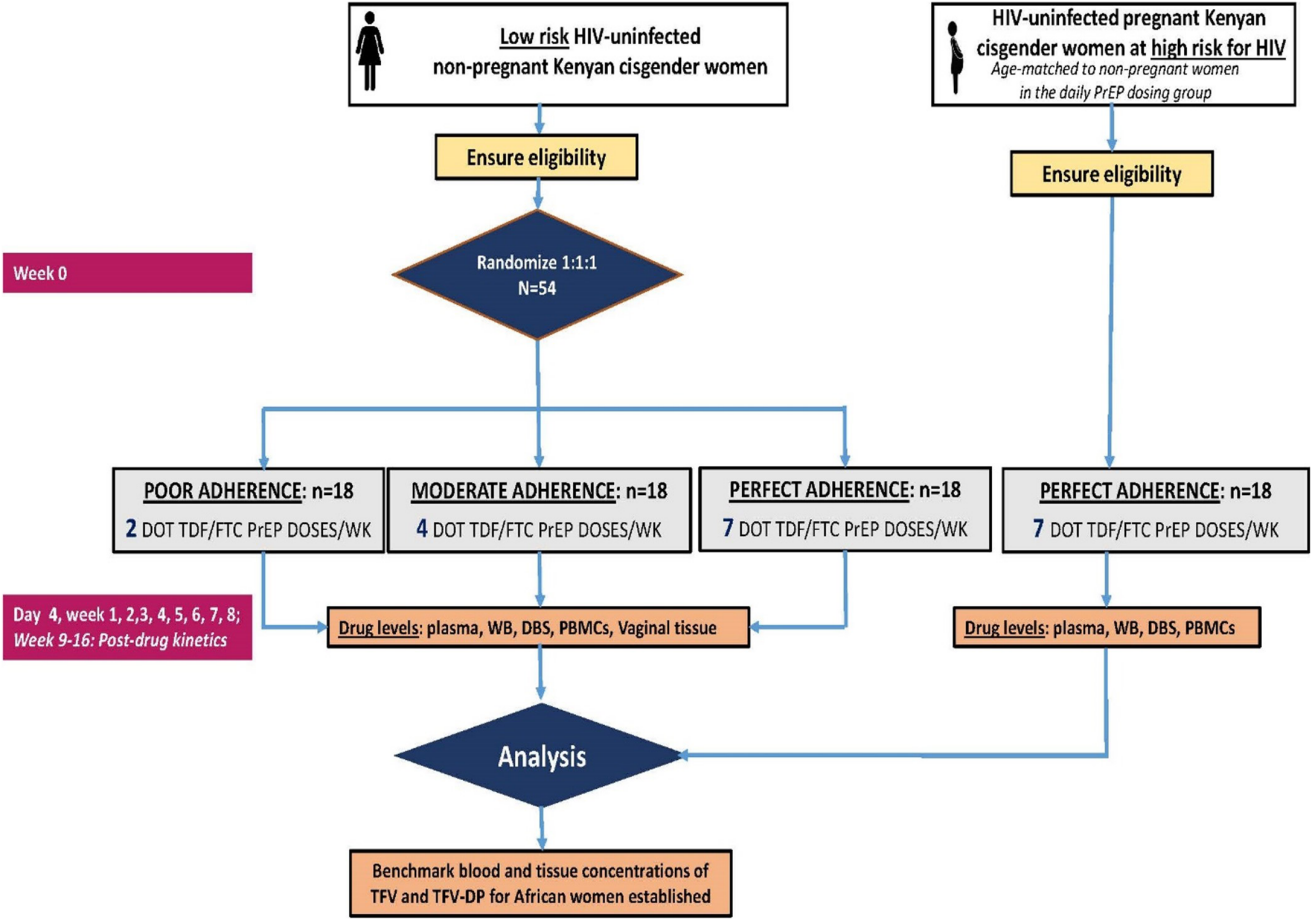
Study schema.

The Women TDF–FTC Benchmark Study is an open-label randomized pharmacokinetic (PK) trial (ClinicalTrials.gov identifier: NCT05057858) (20) designed to define the expected adherence benchmark blood concentrations of TFV–DP for African cisgender women taking directly observed assigned dosing regimens of TDF/FTC (Figure 1). The trial enrolls a target population of healthy, HIV-uninfected, non-pregnant Kenyan cisgender women volunteers aged 18–30 years old to establish benchmark adherence TFV–DP concentration thresholds for TDF-based PrEP. A contemporaneous cohort of pregnant Kenyan cisgender women at an elevated HIV risk was enrolled to define TFV–DP for pregnant women and quantify the impact of pregnancy periods on the benchmark adherence–concentration thresholds.

Forty-five non-pregnant women were randomized 1:1:1 to one of the three dosing frequencies of DOT TDF/FTC (300/200 mg; Truvada®). In Group 1, women received a single tablet of co-formulated TDF/FTC once daily (seven doses per week) for 8 weeks (i.e., perfect adherence arm). In Group 2 or moderate adherence, women received a single tablet of co-formulated TDF/FTC (four doses per week) for 8 weeks. In Group 3 or poor adherence, women received a single tablet of co-formulated TDF/FTC (two doses per week) for 8 weeks. An additional 15 pregnant cisgender women received only daily PrEP dosing (seven doses per week) for 8 weeks.

### Study setting, population, and recruitment

The proposed work took place in Thika, Kenya. The site has extensive experience in conducting large biomedical and PK studies (2, 21–30) and has a long-standing relationship with local public health authorities, HIV prevention advocates, and community leaders. The study enrolled a novel cohort of healthy, HIV-uninfected non-pregnant Kenyan cisgender women volunteers aged 18–30 years at low risk of HIV infection, plus a contemporary cohort of pregnant Kenyan cisgender women at an elevated HIV risk. Volunteers were recruited from the community surrounding Thika, Kenya.

#### Eligibility

Eligible participants for the non-pregnant women cohort were non-pregnant, not breastfeeding, and at low risk of acquiring HIV based on Kenya's national PrEP guidelines (Supplementary Table S1) (31) considering the potential for assignment to a non-daily TDF/FTC dosing schedule. Eligible pregnant women were at 13–26 weeks of gestation upon enrollment, as confirmed by sonographic results, and at high risk for HIV. All eligible cisgender women were aged 18–30 years, were confirmed HIV and hepatitis B surface Ag (HBSAg) negative, had normal kidney function, had no current and past use of HIV PrEP, were willing to receive oral TDF/FTC under DOT for at least 8 weeks, and had no significant medical or psychiatric conditions that would interfere with study participation. All women were provided with study information and gave written informed consent before any study-related procedures were performed. Detailed inclusion and exclusion criteria are summarized in Supplementary Table S2.

### Study procedures

An overview of study visits and the schedule of procedures is presented in Table 1. Participants were screened, enrolled, and followed up for 8 weeks with DOT TDF/FTC and underwent a physical exam. Basic demographics and medical history were obtained at screening. Safety laboratories, including complete blood count, HBSAg, and creatinine clearance, were conducted at screening and during the week 8 visit. HIV rapid testing and urine β-HCG (for non-pregnant participants only) were conducted at screening and at weeks 4 and 8. All enrollments were scheduled on specific days to avoid confusion in implementing the proposed dosing and sampling scheme. All women received a full package of HIV prevention, including risk reduction counseling, condoms, and sexually transmitted infection (STI) testing and treatment at each visit.

**Table 1:** Schedule of study visits and procedures.

Timeline (weeks)	Screening	Entry	DOT PrEP period (weeks)								
			W1		W2	W3	W4	W5	W6	W7	W8
			Day 4	Day 7							
Clinic visits #		I	II	III	IV	V	VI	VII	VIII	IX	X
Procedure											
Informed consent	x	x									
Medical, sexual, contraception, antenatal history review, and vaccine history	x	x	x	x	x	x	x	x	x	x	x
Physical and obstetric exams^a^	x										
Fetal ultrasound for pregnant women^b^	x										
Concomitant medication review	x	x	x	x	x	x	x	x	x	x	x
Dispense backup PrEP medications^c^		x									
Issue study dosing calendar		x									
AE assessment		x	x	x	x	x	x	x	x	x	x
Study bloodwork^d^ (WB, plasma, DBS, PBMC)^e^		x	x	x	x	x	x	x	x	x	x
Blood: hDNA		x									
DBS by fingerstick											x
Study urine^d^		x	x	x	x	x	x	x	x	x	x
Vaginal swab		x	x	x			x				x
Vaginal tissue—non-pregnant only^f^			x	x			x				x
Hair collection							x				x
HIV-1 rapid test^a^	x	[x]					x				x
Safety labs^a,g^	x	[x]									
HBS Ag	x										
STI testing (syphilis, HSV-2, GC, CT)		x									
Urine β-HCG^a^	x	[x]					x				x
DOT^h^		x	x	x	x	x	x	x	x	x	x

[x] indicates if screening occurred more than 30 days before the planned enrollment. The screening and entry visits must be ≤30 days apart.

^a^ Safety labs and/or vitals can be performed as needed at any time.

^b^ If the participant has no record of fetal ultrasound for the current pregnancy, an ultrasound must be conducted before enrollment.

^c^ Study medications are dispensed as described.

^d^ Study bloodwork: WB, DBS, PBMC, plasma, and all available blood cell types; study urine: endogenous molecules such as nucleosides.

^e^ Study blood for pregnant women only at entry, on day 4 (a subset of 50%), at week 1 (a subset of 50%), and at weeks 2, 4, 6, and 8.

^f^ Random subset (50%) on day 4 and the remaining subset (50%) on day 7.

^g^ Protocol-specified safety labs include CBC, HBSAg, and creatinine clearance, in addition to monthly HIV testing and urine pregnancy tests.

^h^ DOT performed if applicable per randomization schedule.

#### Dosing schedule and assessment of adherence

The dosing frequency scheme for non-daily doses was chosen to directly replicate the DOT–DBS (8, 32) study schedule based on the ∼17-day half-life of TFV–DP in DBS (33). The two doses per week arm was taken on Monday and Tuesday, while the four doses per week arm was taken on Monday, Tuesday, Thursday, and Friday. This schedule permits a simultaneous evaluation of the concentrations derived from intermittent and day-by-day dosing. The operationalization of specific days for dose taking can be adjusted prior to study start while maintaining the randomized adherence patterns (frequency of dosing) to permit efficient study operations and minimize the burden on study participants. This is possible due to the long half-life of TFV–DP, which obviates the need for precisely timed collections (one of its advantages). Therefore, any chosen day remains appropriate. For all women, doses were directly observed in person by project staff at the clinic or the participants’ place of convenience. A supply of backup medication (up to a week) was provided if the participant could not complete a visit to the clinic. These doses were directly observed by live WhatsApp video streaming on a smart cell phone. For DOT doses taken via video streaming, the participants were asked to open their mouths afterward to confirm swallowing.

#### Pharmacological sample collection and pharmacokinetic testing

Pharmacological samples collected to measure the respective concentrations of TDF/FTC moieties included blood (for isolation of whole blood, DBS, plasma, and PBMCs), urine, vaginal fluid and tissue, and hair, as listed in Table 1. Blood and urine were collected at study entry, on day 4, and then weekly throughout the 8-week DOT PrEP period. Blood samples were collected from pregnant women on a reduced schedule, comprising at entry, on day 4 (a subset of 50%), at week 1 (a subset of 50%), and at weeks 2, 4, 6, and 8. An additional blood sample for humans was archived from all participants at baseline for future pharmacogenetic studies of drug transporters. Vaginal fluid is collected with a vaginal swab at study entry, day 4, and weeks 1, 4, and 8. Vaginal tissue, obtained via biopsy, was collected only from non-pregnant women on day 4 (a subset of 50%), at week 1 (a subset of 50%), and at weeks 4 and 8. Women who provide vaginal biopsy tissue on day 4 did not have another biopsy at week 1 but provided the remaining samples at weeks 4 and 8. Hair samples were collected at weeks 4 and 8. All samples were collected, processed, and stored according to the standard operating procedures developed by the Colorado Antiviral Pharmacology Laboratory (8).

Drug concentration assays were performed using liquid chromatography–tandem mass spectrometry (LC/MS) methods (1, 34) validated for each biological matrix (e.g., whole blood, DBS, plasma, etc.). The laboratory participates in the NIH-supported Clinical Pharmacology Quality Assurance Program for assay method external review and approval, as well as periodic proficiency testing (35). All assays were validated based on the industry standards for bioanalytical method validation.

### Sample size calculation

The sample size for drug concentration parameters was based on ensuring precision in the estimates to accurately describe the concentration kinetics of TDF/FTC moieties in pregnant and non-pregnant cisgender women and was consistent with multiple DOT PrEP studies done in US populations (8, 32). The precision desired for the mean TFV–DP in DBS at steady state was within ±15% of the true population mean. The mean (±SD) TFV–DP at steady state in HIV-uninfected US adults following directly observed doses of 300 mg TDF/200 mg FTC once daily for 12 weeks was 1,605 ± 405 fmol/punch [coefficient of variation (CV) of 25.2%] (8). Based on this variability and assuming that average TFV–DP results are normally distributed, to be 90% confident that the DBS TFV–DP sample mean was within 15% of the true mean, a sample size of 15 participants per group was required for non-pregnant and pregnant African cisgender women. Thus, the target evaluable sample size (i.e., with all DOT and PK samples) was 15 per group. We enrolled 18 participants per group (i.e., an additional three per group) to account for potential attrition.

### Statistical analysis

Clinical outcomes are described and summarized as overall and by TDF/FTC dosing regimens. Kruskal–Wallis parametric test was used to compare demographics and key study variables by study arms. The primary evaluation focused on the TFV–DP concentrations in DBS and PBMC. Secondary analyses involved TFV–DP or FTC–TP and TFV/FTC concentrations in other biologic matrices. The observed drug concentrations in each biological matrix at each time point are summarized as medians with interquartile range (IQR). Dose proportionality was assessed across the dosing regimens using untimed analyte concentrations for each week of observation through week 8, and the effect of dose on TFV–DP in DBS using two, four, and seven doses per week by DOT was determined. Means, variances, and dose proportionality utilized the measured concentrations at the steady state (Css at 8 weeks). In addition, the following first-order kinetic equation is fitted to the TFV–DP data: Ct = Css × (1 - *e*^−*k*×*t*^), where Ct is the TFV–DP at time *t* (i.e., day 4, weeks 1, 2, 4, 6, and 8), Css is the fitted concentration at the steady-state plateau, and *k* is the fitted elimination rate constant; the half-life is ln(2)/*k*. Under the power model, Css = *a* (dosing)*^b^e*, dose proportionality dictates that 90% CI for *b* is contained within 0.8–1.25. Graphical plots were used to depict the drug concentrations for each participant or pre-specified subgroups (age, trimester). The inter-compartment correlation (e.g., vaginal fluids vs. plasma or PBMC) was assessed using log-transformed concentrations. Non-compartmental PK analysis was used to describe the concentration–time profile for each biologic matrix–analyte pair to determine the area under the curve (AUC). Intersubject and intrasubject variabilities were calculated as CV for the within and across-groups, respectively, using all steady-state estimates. Linear mixed models with random intercepts, 25th percentiles, and receiver operating characteristics curves were used to generate the predicted concentration thresholds for varying dosing frequency (ranging from two to seven doses per week) for each matrix–analyte pair. Adjustments were evaluated for age, study arm, body mass index (BMI), estimated glomerular filtration rate (eGFR), and hematocrit. We quantified the impact of pregnancy on benchmark concentrations by computing the absolute and percent difference of the concentrations in pregnant vs. matched non-pregnant women assigned to seven doses per week.

### Estimating TFV in plasma and intracellular TFV–DP concentrations in DBSs and PBMCs associated with HIV protection for women

Estimation of robust cisgender women-specific adherence–efficacy thresholds requires data that link the pharmacologic measurement of oral PrEP adherence to regular HIV testing in a cohort of women who used PrEP, wherein some women seroconverted and others remained HIV-uninfected. The Partners PrEP Study (2), which has previously been reported, provides a unique opportunity to answer this question. Briefly, the Partners PrEP Study (2) was a phase III, randomized, placebo-controlled trial of daily oral TDF and TDF/FTC PrEP among 4,748 heterosexual HIV-uninfected members (1,780 among women) of HIV serodiscordant couples, which demonstrated definitive HIV protection of PrEP in heterosexual individuals of 67% with TDF and 75% with TDF/FTC and resulted in global PrEP licensure and guidelines for use. The trial was conducted between July 2008 and November 2010 at nine research sites in Kenya and Uganda. The HIV-uninfected partners were randomized 1:1:1 to daily oral TDF, TDF/FTC, or placebo and were followed up monthly for up to 36 months with sexual behavioral assessment, HIV serologic testing, pregnancy testing (for women), risk reduction counseling, and study drug provision. Seroconversions were detected by rapid HIV tests conducted in duplicate at the study sites and confirmed by enzyme immunoassays. After a definitive demonstration of PrEP protection against HIV acquisition, the placebo group was discontinued, the active PrEP arms were continued, and persons initially on placebo were re-randomized (1:1) to the active PrEP arms. An additional 22 HIV infections occurred during this extended follow-up (12 among women), bringing the total number of HIV infections among persons assigned TDF and TDF/FTC to 52, with 29 infections found in women (2, 36, 37).

Within the Partners PrEP Study, a case–cohort was defined with randomly selected participants from the active PrEP arms of the trial (i.e., TDF and TDF/FTC arms) to help closely assess the association between HIV infection and adherence and all HIV seroconverters from the active PrEP (37). For all cohort participants, plasma and WB were obtained and archived at month 1, 6 months thereafter, and at final visits. For each HIV-infected case, plasma and whole blood specimens were obtained at the time of the first evidence of HIV infection in the cases or the nearest visit, as well as longitudinally at other available time points. The Partners PrEP study did not archive DBSs. The assays for measuring TFV–DP and FTC–TP in WB were not developed. Thus, we first used extensively paired WB and DBS in the primary Women Benchmark Study to validate the whole blood–DBS assay for TFV–DP and FTC–TP in the same Colorado Antiviral Pharmacology Laboratory (8). We then tested all available plasma and whole blood samples from the case–cohort for the intracellular TFV–DP concentrations using the same analytic methods employed in the primary DOT study.

The TFV concentrations in plasma and the intracellular concentration of TFV–DP in DBSs or whole blood associated with protection against HIV acquisition were estimated (10, 32). We used Bayesian statistics to combine the observed HIV incidence and prior drug levels on TDF/FTC in the Partners PrEP case–cohort, and, where applicable, information from prior TDF/FTC PrEP studies in women was incorporated to calculate the background HIV incidence in the counterfactual population of Partners PrEP participants had they not been adherent to TDF/FTC and calculate the overall HIV prevention efficacy. Multiple imputation (38) was used to construct a data set with drug concentrations at each visit when an HIV test was performed. The cohort was randomly sampled, making it possible to validly apply multiple imputation (or inverse weight-based methods), consequently satisfying the missing at-random assumption to the case–cohort sampling. The testing scheme ensures validity, even if a high percentage of values at visits at which HIV test was done needs to be imputed. Using the prior distribution of TFV–DP levels (i.e., adherence levels to TDF/FTC), we simulated the posterior distribution for HIV incidence on TFD/FTC PrEP and the background HIV incidence, summarized as posterior medians and 80% posterior credible intervals. Bayesian estimates of the posterior HIV prevention efficacy were calculated by comparing the distribution of HIV incidence on TDF/FTC to counterfactual HIV incidence. Results are summarized as the number of HIV infections averted, number of women needed on TDF/FTC to prevent one new HIV infection, median posterior densities for HIV prevention efficacy, and 95% posterior density credible.

#### Relationship between frequency of PrEP dosing and HIV protection for women

The concentrations of TFV in plasma and TFV–DP in whole blood and DBS from two, four, and seven PrEP doses per week in the present study were analyzed and applied to the primary model fit to the Partners PrEP clinical cohort. The estimated posterior PrEP efficacy and 95% posterior density credible associated with the frequency of PrEP doses per week were approximated using the observed TFV and TFV–DP concentrations for doses per week regimen, with the parameter estimates from the model fitted using multiple imputation with Wald-based CIs. Similarly, the proportions of women in the two, four, and seven doses per week groups who attained TFV and TFV–DP concentrations associated with a range of TFV–DP protective efficacies were computed. We conducted a series of sensitivity analyses to evaluate the robustness of the estimated protective efficacy thresholds, including an analysis that adjusts for condomless sex and other factors used in multiple imputation, allowing drug concentrations below the limit of quantitation to vary uniformly between 0 and 5, using the average drug concentrations from the visit closest to HIV infection with values in the previous 120 days, and bringing drug detection at post-seroconversion time points to the time of the first HIV detection.

#### HIV efficacy among pregnant women

There have been no specific clinical trials assessing the efficacy of HIV TDF/FTC PrEP in pregnant women; hence, it is challenging to establish a concentration–efficacy relationship using the methodologies we proposed for non-pregnant women. Nonetheless, we can analyze and compare the drug concentrations in the whole blood and DBS samples from the pregnant cohort with those observed in non-pregnant women. This will shed light on how pregnancy may influence drug levels in its pharmacologic active site and offer valuable insights into the potential effectiveness of HIV TDF/FTC PrEP in pregnant women.

## Discussion

The adherence–concentration–efficacy thresholds of TFV–DP for MSM have successfully guided the definition of the effective and consistent use of PrEP among MSM. However, many antiretroviral drugs, including TDF-based PrEP, exhibit variable pharmacokinetics (39) due to effects of race, sex, anemia (common in African populations), or physiologic changes during pregnancy (40). Thus, the adherence thresholds derived from US populations may not accurately apply to African cisgender women, and context-specific studies are needed to fill these gaps. In our proposed study, we, for the first time, established the TDF–PrEP adherence–concentration–efficacy thresholds in African cisgender women, including during pregnancy, by using DOT dosing, measuring in multiple biologic matrices, and linking these data to the Partners PrEP Study clinical cohort to define the concentration thresholds associated with HIV protection. Our findings will not only directly improve the interpretation of adherence data and guide optimal PrEP dosing for cisgender women in Africa but also permit an accurate interpretation of ongoing clinical trials of novel HIV prevention products with oral TDF-based PrEP as an active comparator.

Our study has several limitations. First, part of the preliminary data were not from DOT studies and the observed effects might be due to the correlation between MEMS and blood levels and not related to the pharmacologic differences between US male and African women blood levels (12). However, similar findings were reported in African cisgender pregnant women in IMPAACT2009 (14). Second, we have not planned for intensive sampling for individual PK parameters as it is well defined. DBS exhibits a long half-life and is not suited for intensive PK. Nevertheless, plasma steady-state levels, while not pinpointing the mechanism for PK differences, will indicate whether recent PK exposure differs and by how much. Furthermore, the model to quantify concentration efficacy requires us to estimate drug concentrations (i.e., using imputation) in the participants without drug-level testing. A similar approach was used in iPrEx for MSM, and the model has been useful and essential to the field (10).

In conclusion, the results of this work will inform policy aiming at advancing effective PrEP use among cisgender women, including during pregnancy, in Kenya and other sub-Saharan countries.
