# Supplementary material for: Establishing adherence–concentration–efficacy thresholds of TDF–FTC pre-exposure prophylaxis for HIV prevention in African women: a protocol for the Women TDF–FTC Benchmark Study
Source: Front Reprod Health. 2024 May 27;6:1325257. doi: 10.3389/frph.2024.1325257 (PMC11163076; doi:10.3389/frph.2024.1325257)
Supplement: Supplementary file 1 [file Table1.docx]

Supplementary Material

# Supplementary Tables

| **Supplementary Table 1** Kenya national guideline indicators of substantial risk for HIV and indication for PrEP. | | |
| --- | --- | --- |
| - Partner of HIV-infected person not on ART or on ART for <6 months - Has >1 partner of unknown status - Engages in transactional sex - History recent STI (past 6 months) - Recurrent PEP use - Inconsistent or no condom use - Injection drug use | | |
| **Supplementary Table 2** Inclusion and exclusion criteria | | |
| **For all cisgender women** | **Specific for nonpregnant cisgender women cohort** | **Specific for pregnant cisgender women cohort** |
| Inclusion criteria | | |
| - Age ≥18 and $\leq$30 years old - Willing to undergo urine pregnancy tests - Able and willing to provide written informed consent - Test HIV-1 negative (HIV rapid tests) - Normal renal function (estimated glomerular filtration rate >60 mL/min) - Hepatitis B surface antigen negative - No active clinically significant medical or psychiatric conditions that would interfere with study participation - Lack of severe anemia - Willing to use DOT and come to clinic frequently for DOT PrEP for at least 8 weeks - Willing to have home visits for follow up - Has access to an active smartphone to allow off-site observation of dosing or resides in a close location to the clinic to permit home visits if unable to come to the clinic - Intention to stay within the study site's catchment area for at least 8 weeks. - Resides or works in catchment area with high-speed internet coverage to permit video streaming if participant does not reside within a reasonable distance from the clinic | - Not pregnant or breast feeding - At low risk for HIV according to Kenya guideline for PrEP - Willing to be randomized to non-daily PrEP and come to clinic frequently for DOT PrEP - Willingness and ability to be abstinent for at least 7 days after each vaginal biopsy visit | - Evidence of a viable pregnancy with gestational age of 13-26 weeks after the date of conception with sonographic confirmation at screening - At elevated risk for acquiring HIV according to Kenya guideline for PrEP - Willing to use PrEP during pregnancy for HIV prevention at study entry |
| Exclusion criteria | | |
| - Inability to give informed consent - Test HIV-1 negative (HIV rapid tests) or suspected acute HIV infection - Positive HBV surface antigen test at screening - Calculated creatinine clearance < 60 ml/min - Any laboratory value or uncontrolled medical condition that would interfere with the study conditions - Prohibited concomitant medications are: investigational agents (within 30 days of enrollment), aminoglycosides, ganciclovir/valganciclovir, chronic high-dose acyclovir/valacyclovir (>800mg acyclovir or > 500mg valacyclovir for >7 days), cyclosporine, amphotericin B, foscarnet, and cidofovir, and products with same or similar active ingredients as the study medications including TAF®, ATRIPLA®, COMPLERA®, EMTRIVA®, VIREAD®; or drugs containing lamivudine or adefovir, which are close analogs of FTC and tenofovir, respectively. - Current or past use of HIV PrEP - Not willing to have home visits | - Pregnancy or plan to become pregnant in the next 6 months, or unwillingness to use birth control - Current breastfeeding - High risk of HIV infection | - Mother has a known history of any of the following: - Sickle cell anemia (excluding sickle cell trait), chronic bleeding, blood transfusion within the past 120 days (excluding for chronic illness) or other blood dyscrasias - Fetus has a known or suspected major congenital anomaly - Complications in prior pregnancies that would be considered exclusionary |
